# Survey data on factors that influence the adoption of soil carbon enhancing practices in Western Kenya

**DOI:** 10.1038/s41597-020-0374-1

**Published:** 2020-02-10

**Authors:** George Magambo Kanyenji, Stanley Karanja Ng’ang’a, Evan Hartunian Girvetz

**Affiliations:** 1 Department of Agricultural Economics, University of Nairobi, P.O. Box 29053-00625, Kangemi, Nairobi Kenya; 2 International Center for Tropical Agriculture, P.O Box 6247, Kampala, Uganda; 3 International Center for Tropical Agriculture, P.O Box 823-00621, Nairobi, Kenya

**Keywords:** Climate-change mitigation, Agriculture

## Abstract

The data described in this paper were collected in Western Kenya, specifically in Kakamega and Vihiga Counties. The data were collected from 334 households with the aim of assessing factors that facilitate or constrain the adoption of practices that enhance the sequestration of soil carbon. The data were collected through a structured questionnaire that was designed in SurveyCTO. The data were later downloaded from SurveyCTO servers and exported to STATA version 14 for cleaning and analysis. This data can be used by researchers to assess the probability and extent of adoption of specific soil carbon enhancing practices in the two counties of Western Kenya. Additionally, it can be utilized to access the impact of adopting soil carbon enhancing practices on maize and beans yield at both the plot and the farm level.

## Abstract (summary)

**Table:** 

Measurement(s)	Socioeconomic Factors • Access • wealth • plot specific information • agricultural process
Technology Type(s)	Survey
Sample Characteristic - Environment	farm • farm soil
Sample Characteristic - Location	Vihiga County • Kakamega

Machine-accessible metadata file describing the reported data: 10.6084/m9.figshare.11618970

## Background & Summary

Enhancing food security and reducing the poverty rate have been and continue to be vital policy targets for most countries in sub-Saharan Africa (SSA). These challenges are of great concern considering that in SSA the population is expected to double^1^ by 2050 and hence, the demand for food will continue rising sharply^2^. However, overcoming these challenges has been elusive due to weak policy formulation in an ever-changing environment. Additionally, most agricultural land in SSA is faced by low soil fertility due to poor agricultural techniques such as continued mono-cropping resulting in soil degradation^3,4^. At the same time, frequent dry spell and rainfall variability have worsened the situation leading to low agricultural productivity^5^. As a result, in most SSA countries,’ food security policies are geared towards the adoption of sustainable agricultural practices that can aid in enhancing soil fertility and increasing household income^6,7^. Some of the sustainable practices that have been advocated for in SSA include climate-smart agricultural technologies (CSAs) and soil carbon enhancing practices (SCEPs). SCEPs are preferred as they help improve soil fertility and at the same time sequestrating atmospheric carbon^8,9^. Additionally, the implementation of certain practices such as the use of farmyard manure, compost manure, intercropping with legumes is encouraged, as they are considered as low-cost practices^10^ suitable for a majority of the small-scale farmers in SSA who are largely dependent on agriculture and live below the poverty line^11^.

Adoption of SCEPs in Kenya is being advocated for as it can enhance soil fertility over time; however, the adoption rates are still low^12–15^. Published literature indicates that several factors (such as socio-economic factors, external support, and wealth category)^13,14^ can influence the adoption of these technologies. Nevertheless, the influence of plot-specific characteristics has been ignored by most researchers in Kenya. At the same time plot specific characteristics have been established to be critical in influencing the adoption rate of agricultural technologies^16–18^. Motivated by the aforementioned, a survey was conducted in Western Kenya, a high agricultural potential area faced with low soil fertility, soil degradation, land fragmentation due to high population density and relatively high poverty rate^19,20^. In addition, several programs have been implemented in this area to enhance the adoption of these practices. The data contained herein were used to evaluate factors that may facilitate or constrain the adoption of SCEPs. The data contains socioeconomic factors, plot-specific characteristics, external support institution factors, wealth category and information relating to access to specific infrastructures. Additionally, the data takes into account maize and beans yield at the plot level under the different combinations of practices implemented by farmers. The survey tool that was used to collect the data was reviewed and approved by the Internal Review Board (IRB) at the International Centre for Tropical Agriculture (CIAT) before the study was commenced. Additionally, the farmers signed a consent form that indicated that they were willing to participate in the study and would terminate the interview at any point.

The data can be utilized to investigate the probability and extent of adoption of specific SCEPS, reasons behind farmers’ decision to implement particular SCEPs, the challenges they encounter while implementing the practices and the impact of adopting practices – singly or in combination - on maize and bean yield at both plot level and household level.

## Methods

### Data collection

**Fig. 1:**
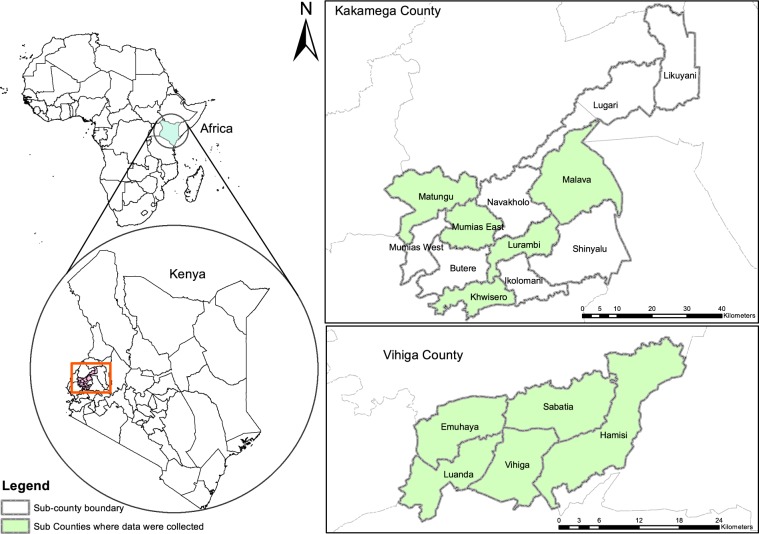
Map of the study area.

The data were collected from Vihiga and Kakamega counties in Western Kenya (Fig. 1). The study area was selected as it is classified as a high agricultural potential area but faced with low soil fertility, soil erosion, soil degradation, and low agricultural productivity. A four-stage multistage sampling technique was utilized to generate the sample. In the first stage, five sub-counties (i.e., Khwisero, Matungu, Malava, Lurambi, and Mumias East) were randomly selected in Kakamega county, while in Vihiga county Vihiga (Emuhaya, Hamisi, Sabatia, and Luanda sub-county) were selected. In the second stage, two wards were selected from each sub-county with the help of the county extension officer. This involved checking the probability of finding farmers that had adopted different SCEPs. In the third stage, from four wards one village and from 6 wards 2 villages were randomly selected in each county. In total 16 villages from each county were selected. In the fourth stage, ten farmers from each village were interviewed, by first picking random farmers from the extension officer list and then snowballing to find the next farmers. The final sample was determined using Eqs. 1 and 2, which resulted in 320 farmers (i.e., 160 farmers from each county). However, to cater to data problems such as missing observation and incompletely filled questionnaires, 14 additional respondents were interviewed, leading to a final sample size of 334 farmers operating 710 plots.

1 $${n}_{0}=\frac{{Z}^{2}pq}{{e}^{2}}$$

2 $${n}_{0}=\frac{1.9{6}^{2}\left(0.5\ast 0.5\right)}{0.05{5}^{2}}=317( \sim 320)$$

where *n*_0_ is the sample size, e is the desired level of precision, *Z*^2^ is standard normal deviation at 95% confidence interval, p is the estimated proportion of an attribute that is present in the population, and q is 1-p.

The data were collected with the help of five trained enumerators who were fluent in both English and Kiswahili. The enumerators undertook a three-day intensive training on how to conduct the interview, understanding the research question and how to key in the data into the tablets since the questionnaire had been digitized into Survey CTO. A pre-test was conducted to test whether the enumerators had mastered the survey tool and whether there were errors that needed to be rectified in the tool before its deployment. Survey CTO was utilized because it helps reduce errors by controlling for missing values and therefore makes it easy to control the quality of data obtained.

## Data Records

The data is available as Stata Data Format (.dta) and across the entire data, missing values are identified with a dot (.), the standard way of representing missing values under the Stata Data Format. All data are stored in the Harvard Dataverse Repository^21^ and are accessible through the Harvard Dataverse Repository online portal. The data are arranged as per the questionnaire that was used to collect the data. The questionnaire was divided into eight sections as shown in Table 1. Section one collected the general information of the study area. Section two collected information relating to the respondents – their name, years in farming and years in farming for the household head). Section three collected data on the household demographic characteristics (i.e., that is the number of people in the household, their age, gender, relation to the household head, occupation, and if they participated in farming activities). Additionally, the section also collected information that helped in determining the household’s wealth category as based on a simple wealth scorecard as well as information relating to household access to different infrastructures such as motorable road, tarmac road, local, livestock and urban markets, electricity, and clinic. Section four contains data relating to farmer’s plot characteristics, perception towards soil erosion, soil type and soil fertility, practices implemented under each plot, main crops grown in the plots, output over the last two growing seasons, inputs utilized, source of labour, livestock, and their participation in crop and livestock markets. Section five contains data associated with farmer’s social capital, while section six and seven established access to credit and access to extension services respectively. Lastly, section eight contains data pertaining to a farmer’s different sources of income. In the survey the household head was targeted; however, in their absence, other household members that were above 18 years were interviewed, provided they had more than five years of farming experience. In this dataset, a household head was defined as the key decision-maker as far as farming was concerned and participated in farming. The different sections of the questionnaire helped us generate the specific variable such as socio-economic factors, plot-specific characteristics, external support, wealth category and access to specific infrastructures factors that may influence the adoption of practices.

**Table 1:** Description of the dataset as per the questionnaire. The questionnaire generated six themes: Socio-economic factors (SOC), Access to infrastructure (INF), Wealth information (WEA), Plot specific information (PLI), Agricultural practices and activities (AGR), and Access to external support services (ESS).

Variable	Theme	Description	Duration Considered
Demographic	SOC	Gender, age, education level, and occupation of the household members.	12 Months
Infrastructure	INF	Distance in walking minutes to the motorable road, tarmac road, local market, nearest livestock market, the urban market, electricity, and clinic.	Current
Wealth Index	WEA	The probability of a household not being poor as guided by 10 questions.	Current
Plot specific Information	PLI	Describes the plot size, the tenure system, manager of the plot, distance of the plot from the homestead, plot’s slope, soil type, and farmers’ perception towards their soil fertility and soil erosion.	Current
SCEPS	AGR	Practice implemented in each plot and the reason behind their implementation and challenges associated with each practice.	Current
Crop Yield	AGR	The three main crops grown by the farmers and their yield in the last two seasons.	Last two cropping season
Crop Market Participation	AGR	Indicates if farmers sold their crop produce and the revenue received.	Last 12 months
Input	AGR	Indicates if farmers utilized chemical fertilizer and inorganic fertilizer. It takes into account the amount utilized, source, and price per unit if purchased.	Last Season
Labour	AGR	Labour sources for farming activities.	Last two cropping season^*^
Livestock	AGR	The livestock owned and purpose of owning the livestock.	Current
Livestock Market Participation	AGR	Indicates if farmers sold their livestock and the revenue received.	Last 12 months
Social Capital	ESS	If the farmers belonged to any farmer group or organization and the purpose of the group.	Last 12 months
Access to Credit	ESS	If a farmer accessed credit, source of the credit, the amount received, and purpose of the credit.	Last 12 months
Access to Extension	ESS	If farmers accessed extension service, source, nature, and terms of provision of the information.	Last 12 months
Sources of Income	WEA	The different sources of income that the household head has.	Last 12 months

*Included the short rain season and long rains season of 2017. The survey was conducted in August 2018.

## Technical Validation

The entire data set described is cross-sectional and was obtained through interviews with farmers. It is common for this type of data to have several problems such as missing information or under or over-reporting. To correct for this SurveyCTO was utilized whereby constraints were embedded in the survey questionnaire to ensure responses to key questions were obtained. The survey employed three techniques to enhance the reliability of data collected. Firstly, before the start of an interview, the farmers were given an overview of questions they were to be asked and in case they objected to giving any sensitive information they had the right to terminate the interview. Secondly, key experts (extension officers) were utilized to validate the values the farmers were giving. Additionally, a focus group discussion with farmers was conducted to get rough estimates of the inputs utilized, land size, and yield. As such their estimated married with the survey results. Additionally, the values were counterchecked with estimates from the literature review, key experts (extension officers) and focus group discussion results.

Among the data described in this paper, there are some variables we would not ascertain fully as they required a farmer’s recollection capability and honesty. However, measures were put in place to enhance the reliability of the variables. With reference to Table 1, variables under demographic, infrastructure, wealth index, SCEPs, labour, livestock, livestock market participation, social capital, access to credit and extension, and source of income are highly reliable and certain. However, other variables that required quantification would be considered as highly certain as described below. Under plot specific information, we are certain of all the variables apart from plot size. Nevertheless, to ensure the reliability of the variable, enumerators would first enquire if the farmer had a title deed and this would be utilized as a measure of the plot size. However, if a farmer had subdivided their land, they would be asked if they know the size of the plot either in meters or acres. The different measurements were then standardized to acres. The study avoided utilizing hectare since most farmers were not familiar with hectare standardization as they were more familiar with acre and meters.

Under crop yield subcategory, the quantity of crop harvested is the only uncertain variable. However, to enhance its reliability, a farmer would quantify their harvest either in bags either 90 kg bag, 70 kg bag, 50 kg bag, in kilograms or other local measurement units that were then converted into kilograms during data cleaning. Under the input section, the only variable the study is not certain of was manure usage, unlike fertilizer usage which is accurate since farmers would recall the quantity they purchased. However, to enhance reliability on the quantity of manure farmers utilized, farmers would specify quantities utilized in local measurements or in kilograms that were later converted into kilograms. Lastly, some farmers would over report selling price for some of their products mainly cash crops such as tea and sugarcane and to correct for this, we replaced the outliers with the mean selling price during the time of the interview as obtained from the Kenya National Bureau of Statistics (KNBS).

## Usage Notes

The data is available in Stata Data Format and can be opened by any Stata program Version 13 and above. However, if using an older version of Stata, Stata provides means of converting the data to be compatible with the previous version. For those who do not have access to Stata software, Statistical Package for Social Sciences (SPSS) software can also be utilized to open the data, under the import tab and specifying that the data is in Stata Data Format (.dta). The questionnaire that was used to collect the data is provided together with the data and will be key to the understanding of the data.

The observations do vary across the files since some households did not participate in the said activity. Additionally, the data in the sub-files is in the long format since the presiding question in the main files was a select multiple. For instance, if a farmer did not access extension, they could not be included in the Access_Extension file. Additionally, if a farmer did access extension but from three sources, they have three entries describing the three sources of extension. Additionally, the data use quite a complex key, computer generated by the Survey CTO data collection software, which was found to be give unique labels preferred to the enumerator pre-assigned key. Lastly, all names of the respondents and their household members were replaced with a 1 to hide their identity and protect their privacy as indicated in the consent form that pseudonyms would be utilized.
